# Effect of preoperative immunonutrition on complications after salvage surgery in head and neck cancer

**DOI:** 10.1186/s40463-019-0345-8

**Published:** 2019-05-31

**Authors:** Simon Andreas Mueller, Catherine Mayer, Beat Bojaxhiu, Carla Aeberhard, Philipp Schuetz, Zeno Stanga, Roland Giger

**Affiliations:** 1 Department of Oto-Rhino-Laryngology, Head and Neck Surgery Inselspital, Bern University Hospital, University of Bern, 3010 Bern, Switzerland; 2 Department of Diabetes, Endocrinology, Clinical Nutrition and Metabolism Inselspital, Bern University Hospital, University of Bern, 3010 Bern, Switzerland; 3 Department of Radiation Oncology Inselspital, Bern University Hospital, University of Bern, 3010 Bern, Switzerland; 4 Department of Endocrinology, Diabetes and Clinical Nutrition University Department of Internal Medicine, Kantonsspital Aarau, 5001 Aarau, Switzerland

**Keywords:** Head and neck cancer, Radiotherapy, Salvage surgery, Immunonutrition, Operative complications

## Abstract

**Background:**

Patients undergoing salvage surgery for recurrent head and neck squamous cell carcinoma are at high risk of postoperative complications due to the adverse effects of radiotherapy on wound healing. Malnutrition is an additional risk factor and we tested the hypothesis that preoperative administration of immunonutrition would decrease complications in this high risk population.

**Methods:**

This single armed study with historical control included consecutive patients undergoing salvage surgery for recurrent head and neck squamous cell carcinoma. We compared outcomes before and after implementation of preoperative immunonutrition and adjusted the regression analysis for gender, age, body mass index, Nutritional Risk Screening (NRS 2002), tobacco and alcohol consumption, tumor localization, tumor stage, and type of surgery. The primary endpoint was overall complications from surgery within a follow-up of 30 days.

**Results:**

Ninety-six patients were included (intervention group: 51, control group: 45). Use of preoperative immunonutrition was associated with a significant reduction in overall complications (35% vs. 58%, fully-adjusted odds ratio 0.30 (95%CI 0.10–0.91, *p* = 0.034). Length of hospital stay was also significantly reduced (17 days vs. 6 days, *p* = < 0.001). No differences in mortality and hospital readmission were found. These results remained robust in multivariate analysis.

**Conclusions:**

In patients undergoing salvage surgery for recurrent head and neck squamous cell carcinoma, preoperative immunonutrition exhibited favorable effects on the complication rate and consequently reduced the length of hospital stay. By improving both tissue regeneration and immune response, immunonutrition may help to improve surgical outcomes in this high-risk population.

## Background

Surgery, radiotherapy (RT) and chemo-radiotherapy (CRT) are mainstays of treatment for head and neck squamous cell carcinoma (HNSCC). Although an effective treatment, (C)RT has significant side effects on local tissues. Fibrosis caused by fibroblast dysfunction and alterations in blood perfusion through microvascular damage lead to impaired wound healing and predispose for local complications [1]. Other side effects such as pain, dysgeusia, xerostomia, vomiting, and inappetence may cause malnutrition, which is an additional independent risk factor for wound healing [2, 3]. Malnutrition, defined as nutrition imbalance leading to weight loss, reduced muscle mass and subcutaneous fat, as well as diminished functional status [4], is a common condition in patients suffering from HNSCC, as these tumors cause aggravation of catabolism and impairment of deglutition through mechanical obstruction or infiltration of the muscles of the tongue and pharynx. Excessive alcohol consumption, a known risk factor for HNSCC, may further impair the nutritional status [5, 6].

Given these unfavorable conditions, patients with HNSCC undergoing salvage surgery for tumor persistence/recurrence or second primaries are at high risk for postoperative complications, with a total incidence of 40–60% described in the literature [7–11]. Complications include wound infections, orocutaneous and pharyngocutaneous fistulas, respiratory insufficiency, pneumonia, and septicemia. Length of hospital stay (LOS) may subsequently be extended and prognosis impaired [12].

Current evidence suggests that perioperative immunonutrition (IN) may reduce complications and LOS after surgery [13]. An intact immune response is crucial for recovery after surgery and especially wound healing. However, it may be compromised by tumor-associated immunosuppression [14] and surgical interventions, which after the initial pro-inflammatory phase cause a proportionate immunosuppressive phase [15]. Malnutrition leads to a deficiency of essential nutrients needed for cell replication, such as nucleotides, amino acids, fatty acids, bases, phosphate and metal ions, and weakens the immune system additionally. The concept of perioperative IN is to provide the essential nutrients to promote an adequate immune response after surgery. Immunonutrition are medical dietary formulas designed to provide the essential nutrients for an adequate immune reaction during medical treatment, such as surgical interventions. Although the bulk of clinical data stems from trials in abdominal surgery [16, 17], a few studies including our own recently published analysis of 411 patients are supporting this concept for surgery in HNSCC [12, 18–22]. However, none of these studies have focused on the especially vulnerable patients undergoing salvage surgery after (C)RT, and the aim of this study was to evaluate the effect of preoperative IN on postoperative complications this high risk population.

## Methods

### Study design and population

The study was conducted in accordance with the 1957 Declaration of Helsinki, and the protocol was approved by the Ethics Committee of the Canton of Bern, Switzerland (Ref. no. 256/2015).

This is a single armed study with a historical control cohort and included patients undergoing salvage surgery for persistent/recurrent or second primary HNSCC after curatively intended RT, CRT or RT with concomitant immunotherapy (Cetuximab) for tumors arising in the oral cavity, oropharynx, hypopharynx, and larynx, as well as carcinoma of unknown primary (CUP) of the neck. All tumor stages were included. Conformal 3D, intensity modulated or volumetric modulated arc radiotherapy techniques were used for initial treatment. Patients were excluded if the (C)RT did not affect the operative field of salvage surgery with more than 50 Gray. Diabetes or treatment with immunosuppressive drugs did not lead to exclusion. The intervention group receiving IN included consecutive patients treated between July 2012 and September 2016. Immunonutrition was introduced in January 2012, but compliance was not monitored during the first 6 months and therefore, patients treated in this period were excluded. The control group who did not receive IN included consecutive patients treated between July 2008 and December 2011. Both groups were treated according to internal guidelines of our tertiary university hospital by multiple surgeons of the same surgical team.

All evaluated surgical salvage procedures were performed with curative intent and based on institutional tumor board decision.

Preoperative body mass index (BMI) and nutritional status were assessed using the Nutritional Risk Screening 2002 (NRS 2002) [23] score. NRS 2002 takes into account patients’ weight loss, BMI, food intake, severity of disease, and age. The score ranges from 0 (no nutritional risk) to 6 (high nutritional risk), and in a non-study setting, nutritional support is recommended for patients with scores ≥3. For patients receiving IN, these parameters were assessed prior to IN administration. Socio-demographic data, concomitant diseases and risk factors (smoking, alcohol overconsumption), and tumor specific data were recorded from hospital charts. All RT plans were reviewed to assess total radiation dose to the tissue in the operative field. The TNM system (International Union against Cancer UICC 7th edition) [24] was used for staging of disease.

### Immunonutrition regimen

The IN used in this study was Oral Impact® (Nestlé Health Science, Vevey, Switzerland), which has been used in various studies including healthy as well as cancer patients and has shown to be safe [25, 26]. One unit (74 g powder to be dissolved in 250 ml of water) provides 300 kcal, and contains 16.8 g protein, 8.3 g fat, and 40.2 g carbohydrates. The sip feed is enriched with omega-3 fatty acids (1.0 g/unit), arginine (3.8 g/unit), RNA-nucleotides (0.39 g/unit) and soluble guar fiber (3 g/unit). Patients in the intervention group received 3 units per day for 5 days before surgery. For monitoring of compliance, patients tagged each consumed dose on a form collected on the day of surgery, and missing data were completed via telephone call.

### Endpoints

The primary endpoint was defined as overall wound complications within the first 30 days after surgery. The total number of patients with wound complications was recorded, and wound complications were also categorized into the following groups: wound dehiscence, abscess, fistula, hematoma, hemorrhage, seroma, and flap necrosis. Additionally, the severity of local complications was graded according to the Buzby classification [27]. Furthermore, the Dindo classification [28] was applied, which captures and grades both local and systemic complications. Complications were recorded regardless whether they occurred during or after hospitalization, as long as they arose within the first 30 days after surgery. Length of total hospital stay was defined as the total number of days spent in hospital after surgery, including readmissions. General criteria for discharge were mobilization, no ongoing wound issues, sufficient nutritional intake, and assured aftercare. Data were recorded retrospectively via chart review by a postgraduate and checked for accuracy by senior staff member of the Department of Oto-Rhino-Laryngology, Head and Neck Surgery.

For subgroup analysis, we defined *extensive surgery* as open tumor resection with or without neck dissection and with or without flap reconstructions. *Restricted surgery* was defined as transoral resection with or without neck dissection or neck dissection alone.

Compliance to the IN regimen was measured as the percentage of the total planned intake that was administered correctly, and four subgroups were formed (0–24%, 25–49%, 50–74%, 75–100%).

### Statistical analysis

Categorical variables are presented as percentages (numbers), and continuous variables are presented as medians (interquartile range, IQR) or 95% confidence intervals (CI) where applicable. The chi-square (Wald) test was used for frequency comparisons and two-group comparisons were assessed with the Mann-Whitney U-test. Univariate and multivariate regression analyses were applied to determine the effect of IN on the primary endpoint and secondary endpoints. To better assess IN’s impact on the endpoints in this non-randomized setting and to account for possible confounders, we calculated a multivariate regression model adjusted for gender, age, body mass index, NRS 2002 [23], tobacco and alcohol consumption, tumor localization, tumor stage, type of surgery, flap reconstruction and comorbidities. To evaluate subgroup effects, we assessed effect modification by including interaction terms into our statistical models. Results were considered statistically significant if *p* < 0.05. Correlations were calculated using Pearson’s or Spearman’s correlation coefficients when needed. The statistical analysis was performed on IBM SPSS Statistics for Windows (IBM Corp., 2010, Version 19.0. Armonk, New York, USA) and STATA 12.1 (StataCorp LP, 2011, Texas, USA) software.

## Results

One hundred and five patients were evaluated, of which nine were excluded because RT did not affect the operative field, leaving 96 patients undergoing salvage surgery for persistent/recurrent HNSCC or second primaries after (C)RT. Fifty-one received IN, while the control group included 45 patients. Immunonutrition was administered orally in 41 patients (80%) and via previously inserted percutaneous endoscopic gastrostomy tube in 10 patients (20%). Socio-demographic and clinical characteristics are summarized in Table 1.

**Table 1:** Socio-demographic and medical characteristics

Characteristics	Total *n* = 96	Control *n* = 45	Intervention *n* = 51	*p-*value
Mean age, years (SD)	65.4 (10.56)	65.4 (10.0)	65.5 (11.1)	0.97
Gender				
Male	76 (79%)	35 (78%)	41 (80%)	0.75
Female	20 (21%)	10 (22%)	10 (20%)	
Smoking				
No	24 (25%)	11 (24%)	13 (25%)	0.11
Active	33 (34%)	20 (44%)	13 (25%)	
Ceased	39 (41%)	14 (31%)	25 (49%)	
Smoking (pack years), median (IQR)	45 (30, 60)	47.5 (30, 68)	42.5 (38, 60)	0.88
Alcohol				
No	43 (45%)	20 (44%)	23 (45%)	0.99
Active	42 (44%)	20 (44%)	22 (43%)	
Ceased	11 (11%)	5 (11%)	6 (12%)	
Alcohol (glass/week), median (IQR)	14 (6,42)	18 (4,70)	14 (6,40)	0.53
Body mass index at admission, mean (SD)	23.29 (3.95)	23.84 (3.84)	22.80 (4.03)	0.21
Nutritional Risk Screening 2002^24^				
Score< 3	78 (81%)	40 (89%)	38 (75%)	0.21
Score≥ 3	18 (19%)	5 (11%)	13 (25%)	
Comorbidities				
Diabetes mellitus	10 (10%)	6 (13%)	4 (8%)	0.38
Hepatopancreatic disease	6 (6%)	2 (45)	4 (8%)	0.49
Cardiovascular disease	57 (59%)	25 (56%)	32 (63%)	0.47
Pulmonary disease	16 (17%)	7 (16%)	9 (18%)	0.78
Other diseases	25 (26%)	8 (18%)	17 (33%)	0.08
Immunosuppression (drug induced)	1 (1%)	0 (0%)	1 (2%)	0.35
Type of tumor				
Persistence/recurrence	76 (79%)	34 (76%)	42 (82%)	0.41
Second primaries	20 (21%)	11 (24%)	9 (18%)	
Localization				
Oral cavity	30 (31%)	18 (40%)	12 (24%)	0.35
Oropharynx	20 (21%)	7 (16%)	13 (25%)	
Hypopharynx	9 (9%)	5 (11%)	4 (8%)	
Larynx	25 (26%)	11 (24%)	14 (27%)	
Lymph node recurrence	12 (13%)	4 (9%)	8 (16%)	
UICC-stage of recurrent/persistent tumors and second primaries				
I	21 (22%)	11 (24%)	10 (20%)	0.55
II	25 (26%)	12 (27%)	13 (25%)	
III	21 (22%)	7 (16%)	14 (27%)	
IV	29 (30%)	15 (33%)	14 (27%)	
Type of surgery				
Restricted	44 (46%)	17 (38%)	27 (53%)	0.14
Extensive	52 (54%)	28 (62%)	24 (47%)	
Flap reconstruction				
No	58 (60%)	30 (67%)	28 (55%)	0.24
Yes	38 (40%)	15 (33%)	23 (45%)	
Tracheostomy				
Yes	25 (11%)	15 (33%)	10 (20%)	0.16
No	71 (89%)	30 (66%)	41 (80%)	
Long-term tracheostomy after surgery > 30 days				
Yes	11 (11%)	7 (16%)	4 (8%)	0.33
No	85 (89%)	38 (84%)	47 (92%)	
Feeding modality before surgery				
Oral	79 (82%)	38 (84%)	41 (80%)	0.42
NG tube	1 (1%)	1 (2%)	0 (0%)	
PEG tube	16 (17%)	6 (13%)	10 (205)	
Feeding modality after surgery				
Oral	29 (30%)	12 (27%)	17 (33%)	0.28
NG tube	43 (45%)	24 (53%)	19 (37%)	
PEG tube	24 (25%)	9 (20%)	15 (29%)	
Peri−/postoperative antibiotic treatment				
Yes	92 (96%)	43 (96%)	49 (96%)	0.90
No	4 (4%)	2 (4%)	2 (4%)	
Duration (days) of peri−/postoperative antibiotic treatment, median (IQR)	12 (10, 16.5)	12 (10, 19)	13 (11, 15)	0.82
Maximum RT dose to operative field (Gray), median (IQR)	70 (66,72)	67 (61, 72)	72 (66, 72)	0.09
Time RT to surgery (days), median (IQR)	524 (231,1645)	874 (311, 1993)	436 (202, 1276)	0.05

*IQR* interquartile range, *NG* nasogastric, *PEG* percutaneous endoscopic gastrostomy, *RT* radiotherapy, *SD* standard deviation, *UICC* Union of International Cancer Control [24]

Both groups showed similar distributions of sex, age, risk factors, and preoperative BMI. The proportion of patients with an NRS 2002 [23] ≥3 was higher in the intervention group, but the difference was not statistically significant. Flap reconstruction was preformed in 23 patients who received IN (45%, 19 pedicled and 4 free flaps) and 18 patients of the control group (33%, 11 pedicled and 4 free flaps; *p* = 0.24). Number of tracheostomies, feeding modality and administration of peri−/postoperative antibiotics were not statistically significant different between the two groups (Table 1).

Complications are shown in Table 2. The total number of patients suffering any complications was significantly lower in the group receiving IN (35% vs. 58% in the control group, *p* = 0.027). As shown in Table 2, this reduction remained robust (adjusted OR 0.28, *p* = 0.049) in the multivariate model adjusted for socio-demographics, risk factors, tumor characteristics, type of surgery, flap reconstruction, and comorbidities. A decrease was observed in all subcategories of complications (wound dehiscence, wound abscess, fistula, and hematoma/hemorrhage/seroma), but the differences on subcategory level were not statistically significant. The total number of patients with local complications was not statistically different between the two groups (control group: 22% vs. intervention group: 18%, *p* = 0.57).

**Table 2:** Effects of immunonutrition on postoperative complications and multivariate analysis

Endpoint	Control	Intervention	*p-*value	Multivariate model^b^	
	*n* = 45	*n* = 51		Adjusted OR (95%CI)	*p-*value
Number of patients with local or systemic complications	26 (58%)	18 (35%)	0.027	0.28 (0.08 to 1.00)	**0.049**
Local complications^a^					
Wound dehiscence	9 (20%)	7 (14%)	0.41	0.60 (0.03 to 1.44)	0.11
Wound abscess	7 (16%)	6 (12%)	0.59	0.24 (0.02 to 3.09)	0.28
Fistula	8 (18%)	5 (10%)	0.25	0.64 (0.03 to 14.1)	0.77
Local hematoma, hemorrhage, seroma	5 (11%)	5 (10%)	0.83	0.20 (0.02 to 2.08)	0.18

^a^More than 1 complication possible per patient. ^b^The multivariate model is adjusted for gender, age, body mass index, Nutritional Risk Screening 2002 [23], smoking habit, alcohol habit, tumor localization, tumor stage, type of surgery, flap reconstruction, and comorbidities. *P*-values shown in bold indicate significance

The severity of the complications as graded by the Buzby [27] and Dindo [28] classification did not show significant differences between the two groups (Table 3). There were no fatalities in either group within the first 30 days after surgery.

**Table 3:** Number and grading of complications according to the Buzby [27] and Dindo [28] classifications

Grade	Definition	Control *n* = 45	Intervention *n* = 51	*p-*value
Buzby classification (local complications) [27]				
I	Redness, swelling, wound not opened	0 (0%)	1 (2%)	0.27
II	As Grade I, but wound opened, dehiscence	5 (11%)	3 (6%)	
III	Pus visible in wound	5 (11%)	3 (6%)	
IV	Fasciitis with surgical debridement	0 (0%)	2 (4%)	
Dindo classification (local and systemic complications) [28]				
I	Any deviation from the normal postoperative course without the need of pharmacological treatment or surgical, endoscopic, or radiological intervention	8 (18%)	5 (10%)	0.52
II	Requiring pharmacological treatment	11 (24%)	5 (10%)	
III	Requiring surgical, endoscopic, or radiological intervention	5 (11%)	7 (14%)	
IV	Life-threatening complication requiring intensive care management	2 (4%)	1 (2%)	
V	Death of patient	0 (0%)	0 (0%)	

**Fig. 1:**
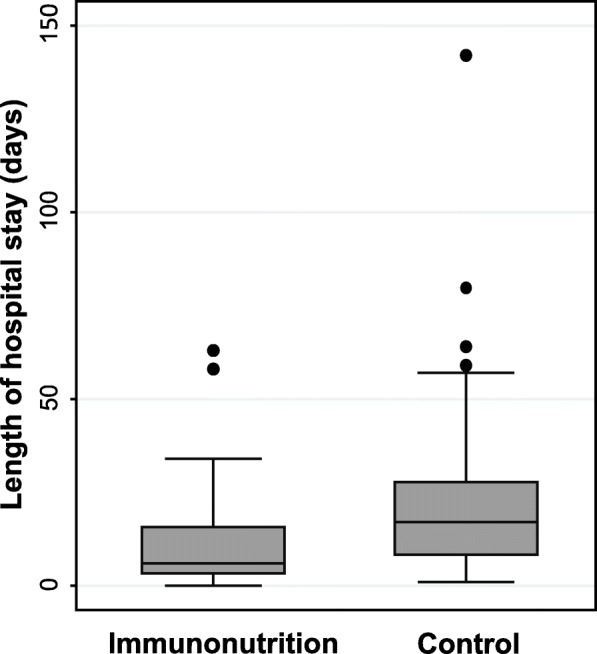
Length of hospital stay. Box plot comparing length of hospital stay between the group that received immunonutrition before salvage surgery and the control group (*p* < 0.001)

Secondary outcome analysis showed a significant reduction in LOS in the IN group (adjusted difference − 11.36 days, (95%CI − 20.08 to − 2.63), median 6 (mean 11.5) days vs. 17 (mean 24.2) days in the control group, *p* = < 0.001), while the rate of readmissions was similar in both groups (Table 4 and Fig. 1). Total or partial flap necrosis and surgical interventions due to complications were similar in both groups. These results remained robust after multivariate analysis (Table 4). Table 5 shows compliance in the IN group; 84.3% of patients took over 75% of the prescribed nutrition. The subgroups were too small to allow a statement on the correlation between compliance and outcome parameters.

**Table 4:** Effect of immunonutrition on length of hospital stay and other secondary outcome parameters

Endpoint	Control	Intervention	*p-*value	Multivariate model^a^	
	*n* = 45	*n* = 51		Adjusted OR (95% CI)	*p-*value
Total LOS, median (IQR)	17 (8, 28)	6 (3, 16)	< 0.001	−11.36 (−20.08 to − 2.63)	**0.011**
Flap total or partial necrosis	1 (7%)	2 (9%)	0.83	–	–
Surgery due to complications	8 (18%)	10 (20%)	0.82	0.45 (0.06 to 3.44)	0.44
Readmissions	4 (9%)	4 (8%)	0.85	0.17 (0.01 to 3.82)	0.27
Mortality within 30 days	0 (0%)	0 (0%)	–	–	–

^a^The multivariate model is adjusted for gender, age, body mass index, Nutritional Risk Screening 2002 [23], smoking habit, alcohol habit, tumor localization, tumor stage, type of surgery, flap reconstruction, and comorbidities. *P*-values shown in bold indicate significance. *LOS* length of hospital stay, *IQR* interquartile range

**Table 5:** Compliance with planned intake of immunonutrition

R_RI/TPI_^a^	No. of patients
0–24%	2 (3.9%)
25–49%	1 (2.0%)
50–74%	5 (9.8%)
75–100%	43 (84.3%)

^a^ Ratio = $$ \frac{\mathrm{Real}\ \mathrm{intake}}{\mathrm{Theoretically}\ \mathrm{planned}\ \mathrm{intake}} $$ ×100

## Discussion

To our knowledge, this is the first study investigating the effect of preoperative IN on short-term outcomes after salvage surgery in previously irradiated patients with HNSCC. Our results show a significant reduction in the number of patients suffering complications (35% vs. 58%), in the group with IN intake before salvage surgery. Compared to other studies on the incidence of complications after salvage surgery without IN, who reported rates of 41–61% [7–11], complications in the IN group (35%) in our study were low.

Our results also showed that patients receiving IN had significantly lower LOS (6 days vs. 17 days), which is in line with several other authors for both gastrointestinal and head and neck surgery [16, 17, 22, 29–32]. This reduction may be attributable in part to the lower rate of complications in the IN group, but seems out of proportion compared to the reduction of complications. Other possible underlying causes for prolonged LOS such as age (leading to slower rehabilitation), tumor localization, type of surgery and flap reconstruction in particular, as well as comorbidities, were considered in the multivariate analysis, but the significance of the reduction in LOS remained robust. Another possible factor that was not included in the multivariate analysis is the slightly higher rate of permanent tracheostomies in the control group (Table 1), but the tracheostomy rate only varied insignificantly (*p* = 0.16), and we consider it unlikely to be the driving force behind longer LOS in the control group. Complications due to PEG tube insertions could also potentially prolong hospital stay, but while the rate of postoperative feeding tube (PEG and NG tube) was similar in both groups, PEG tubes were actually slightly less common in the control group (Table 1), and therefore not associated with the longer LOS of the control group. More likely, the results on LOS may be partially biased because of the historical nature of the control cohort and in particular, the introduction of the diagnosis related groups system in Switzerland (SwissDRG) in 2012. SwissDRG penalizes prolonged hospital stays and has led to optimization of the discharge process of patients. This may have contributed partially to shorter LOS in the IN group, who were treated after 2012.

The effect of IN on surgical outcomes has been studied more extensively in gastrointestinal surgery, and two large meta-analyses on the impact of IN were published in 2012, including a combined 29 controlled randomized trials [16, 17]. Both studies concluded that IN reduces infectious and non-infectious postoperative complications and LOS. Guidelines of both the European and American Society of Parenteral Nutrition therefore recommend IN for at least all malnourished patients undergoing major abdominal surgery [33, 34].

Scientific data on the effects of IN in head and neck surgery is much less definite, primarily due to the limited number of large prospective randomized controlled trials [35]. Moreover, several of the so far published trials reported significant problems with compliance to the prescribed diets [20, 22]. In their systematic review of 10 trials investigating the effect of arginine-based IN on postoperative outcomes in head and neck cancer, Stableforth et al. [35] report that LOS was reduced by 3.5 days in groups receiving IN compared to standard formula or control. The reasons for this reduction remain unclear since it does not necessarily correlate with the complication rates of the analyzed trials. Several trials reported a lower rate of infectious complications [20, 22], wound infections/complications [22, 36], and fistula formation [29–31, 37]. These findings are contested by other trials which found no differences in postoperative complications at all [32, 38]. None of these studies report results for patients previously treated with RT. In their prospective randomized, double-blind study, Falewee et al. [22] included patients who had received previous RT when it was concluded more than 1 year before the trial, but no subgroup analysis was reported. The other studies focusing on HNSCC either excluded patients with previous RT [21] or did not specify whether such patients were included [12, 29–32, 36–38].

Radiotherapy increases the risk of wound healing problems. Microvascular damage and activation of coagulation lead to reduced blood flow in irradiated tissue [1, 39]. The resulting hypoxia induces proliferation of subendothelial connective tissue in small arteries leading to narrowing and obliteration of the vessel lumen, aggravated additionally by thrombosis [40, 41]. The second critical factor is fibrosis, which is particularly strong in the cutis and subcutis [1], where dysfunctional fibroblasts produce excess extracellular matrix that irreversibly replaces normal elastic and collagen fibers and adipose tissue. The resulting tissue is thus hypoxic, hypovascular and hypocellular and the overlying skin suffers atrophy marked by thinning and loss of adnexal structures [41]. Hypovascularity and hypoxia continue to impede physiological wound healing even years after RT and make the affected tissues more susceptible for bacterial infections [42]. This risk is further elevated in case of arginine deficiency, which weakens the immune response by inhibiting T-cell proliferation. The underlying mechanism is a reduction in a ζ-chain component of the T-cell receptor, which is also reduced by certain cancers and after surgery [43]. Furthermore, in activated myeloid cells, arginine is metabolized by the enzymes inducible nitric oxide synthase (iNOS) and arginase 1. The former generates nitric oxide NO, which is indispensable in fighting infections. The latter produces ornithine, a crucial precursor in collagen synthesis [44–46]. Omega 3 fatty acids may inhibit collagen deposition, minimize scar formation and reduce wound infections [25]. Thus, deficiencies in arginine and omega 3 fatty acids impair the immunological response to infections as well as the wound healing process, and potentiate the long term tissue effects of RT. Given this background, it seems conceivable that supplementation of arginine and omega 3 fatty acids may have a marked impact in preventing complications of patients undergoing salvage surgery after RT.

### Limitations

This study is limited by its retrospective character, the fact that it used a historical control group, and the limited number of patients. To account for the possible confounders inherent to this study design, we conducted the multivariate analysis, in which the results remained robust (Tables 2 and 3). As discussed earlier, the disproportionate reduction in LOS in the group receiving IN may be partially biased due to the historical nature of the control cohort and the introduction of the diagnosis related groups system in Switzerland (SwissDRG) in 2012, leading to optimization in the discharge process of patients.

Interestingly, the median maximum dose of radiation to the operative field was higher in the IN group (*p* = 0.09, Table 1), and the median interval between radiation and salvage surgery was shorter (*p* = 0.05, Table 1). However, the maximum radiation dose to any part of the tissue within operative field is not representative of the total or mean dose to this tissue. The exact calculation of the mean radiation dose to the operative field is inherently difficult if not impossible, since salvage surgery is not performed along radiation fields and will always involve areas of tissue irradiated at variable doses (or not at all). Any conclusion on the correlation of IN, radiation dose, and complication rates based on our data would therefore be highly speculative.

## Conclusions

In patients undergoing salvage surgery for persistent/recurrent HNSCC and second primaries after initial RT, preoperative IN is associated with a reduction of the overall complication rate and consequently decreased the LOS. Our results suggest that preoperative IN may be of particular benefit in this high-risk population, as its effects may improve tissue regeneration and immune response, which are two main reasons for impaired wound healing and infectious complications after (C)RT. Prospective randomized trials are necessary to deliver definite evidence to justify the systematic perioperative use of IN in surgery for HNSCC and based on the results of our study, we strongly recommend stratified randomization.

## Abbreviations

BMI: Body mass index
CRT: Chemo-radiotherapy
HNSCC: Head and neck squamous cell carcinoma
IN: Immunonutrition
LOS: Length of hospital stay
NG: Nasogastric (feeding tube)
NRS: Nutritional risk screening
PEG: Percutaneous endoscopic gastrostomy
RT: Radiotherapy
